# Drainage of Complex Walled-Off Pancreatic Fluid Collections in LAMS Era: A Multicenter Study

**DOI:** 10.1155/2022/9250370

**Published:** 2022-10-29

**Authors:** Thanawat Luangsukrerk, Kamin Harinwan, Stanley Khoo, Pradermchai Kongkam

**Affiliations:** ^1^Division of General Internal Medicine, Department of Medicine, Faculty of Medicine, Chulalongkorn University and King Chulalongkorn Memorial Hospital, Thai Red Cross Society, Bangkok, Thailand; ^2^Excellence Center for Gastrointestinal Endoscopy, King Chulalongkorn Memorial Hospital, Thai Red Cross Society and Division of Gastroenterology, Department of Medicine, Faculty of Medicine, Chulalongkorn University, Bangkok, Thailand; ^3^Division of Gastroenterology, Department of Medicine, Faculty of Medicine, Phramongkutklao Hospital, Phramongkutklao College of Medicine, Bangkok, Thailand; ^4^Gastroenterology and Hepatology Unit, Department of Medicine, Faculty of Medicine, University of Malaya, Kuala Lumpur, Malaysia; ^5^Division of Hospital and Ambulatory Medicine, Department of Medicine, Faculty of Medicine, Pancreas Research Unit and Tropical Medicine Cluster, Chulalongkorn University, Bangkok, Thailand

## Abstract

**Background:**

The lumen-apposing metal stent (LAMS) has been increasingly used for EUS-guided drainage of symptomatic walled-off pancreatic fluid collection (WOPFC) in recent years. Nevertheless, some WOPFCs may require additional drainage methods including another LAMS as a result of complexity of the lesions. This current study aimed to compare clinical parameters of patients with complex WOPFC requiring LAMS with additional methods (complex WOPFC: group A) versus single LAMS alone (noncomplex WOPFC; group B).

**Method:**

Medical records of patients with complex (group A) versus noncomplex WOPFCs (group B) were reviewed and compared in three centers in Thailand and Malaysia, between January 2016 to December 2020.

**Result:**

31 patients with WOPFCs were recruited. 6 of 31 (19%) patients were in group A. Multivariate analysis showed that the maximal diameter of WOPFCs in group A was significantly larger than that of group B (18 ± 6 versus 13 ± 3 cm in diameter, respectively, *p* = 0.021). Solid component proportion was higher in group A versus B (35.8% versus 17.8%, respectively, *p* = 0.025). The prevalence of pancreatic duct leakage was significantly higher in group A (67% versus 20%, *p* = 0.23). The need of direct endoscopic necrosectomy (DEN) and the number of DEN sessions were higher in group A versus B (100% vs. 48%, *p* = 0.020 and 3.5 vs 0 *p* = 0.031, respectively).

**Conclusions:**

Complex WOPFC had larger diameter of lesions, higher proportion of solid component, higher prevalence of pancreatic duct leakage, and higher number of DEN is required than group noncomplex lesions. *Trial Registration.* This trial is registered with TCTR20180223004.

## 1. Introduction

Acute pancreatitis may lead to symptomatic pancreatic fluid collection that requires further treatment. According to the revised Atlanta criteria, walled-off PFC (WOPFC) is classified to pseudocyst and walled-off pancreatic necrosis (WON) [1]. From our previous data, 11.3% of patients with pancreatitis have WOPFCs [2]. On the condition that WOPFCs are infected or symptomatic, drainage is required. Endoscopic ultrasound (EUS) is the first drainage method of choice for WOPFCs in comparison with surgical and percutaneous drainage [3].

The specifically designed lumen-apposing metal stent (LAMS) provides innovative management of WOPFCs [4, 5]. LAMSs are used for both EUS-guided drainage of symptomatic WOPFCs (EUS-PFC) and facilitation of direct endoscopic necrosectomy (DEN) in needed cases [6]. In general, one LAMS has 86–91% to accomplish EUS-PFC drainage with or without DEN [7]. Nevertheless, some WOPFCs may require more than one LAMS as a result of the complexity of the lesion including high viscosity of collected fluid and large amount of necrotic debris. To achieve adequate drainage, percutaneous drainage, surgical debridement, and multiple gateways endoscopic drainage could be used as adjunctive therapeutic modalities for complex WOPFCs.

In the current study, we aimed to compare demographic and clinical relevant data in patients with WOPFCs requiring EUS-PFC between complex ones drained with LAMS (s) plus additional drainage methods and noncomplex ones drained with only a single LAMS. Perhaps, results may help endoscopists to prepare adequate resources before the initiation of drainage procedures.

## 2. Materials and Methods

### 2.1. Study Design

This is a multicenter observational study. The study was conducted in three large referral hospitals including (1) King Chulalongkorn Memorial Hospital, Chulalongkorn University, Bangkok, Thailand, (2) Phramongkutklao Hospital, Phramongkutklao College of Medicine, Bangkok, Thailand, and (3) the University of Malaya, Kuala Lumpur, Malaysia. The medical records of recruited patients between January 2016 to December 2020 were retrieved and reviewed.

### 2.2. Patients

Patients with WOPFCs who underwent EUS-PFC with at least one LAMS in the above three hospitals during the study were recruited. Patients with WOPFCs requiring LAMS with additional drainage methods including additional LAMS, percutaneous drainage, or surgical debridement were categorized as “complex WOPFCS” and classified as group A, whereas ones requiring only single were grouped as “noncomplex WOPFCS” or group B. All baseline patients' data including endoscopic procedures, radiologic examinations, and other modalities of WOPFCs drainage in the 6-month period after EUS-PFC were retrieved, recorded, reviewed, and compared between both groups.

### 2.3. Procedure and Devices

The EUS-PFC drainage protocol in this current study is an endoscopic step-up approach [8]. The selection of routes of EUS-PFCs, between trans-gastric versus trans-duodenal approach, depends on the appropriate position of EUS access. A LAMS (10, 15-mm, AXIOS™, Xlumena Inc., Mountain View, CA, USA, or 10, 16-mm, SPAXUS™, Taewoong Medical co., Ilsan, Korea, or 16-mm NAGI™, Taewoong Medical co., Ilsan, Korea) was initially used for creating cystogastrostomy and/or cystoduodenostomy. LAMS with a 15-mm luminal diameter or larger are defined as large-diameter LAMS. Technical success was defined as the successful placement of LAMS and/or drainage catheter in the desired position. The clinical success rate was defined as the maximal diameter of lesions reduced by more than 50% without clinical symptoms requiring additional drainage. In cases requiring additional drainage, procedures included percutaneous drainage, multiple LAMSs, or surgical debridement. All LAMSs were removed after two weeks of placement to prevent bleeding [9].

### 2.4. Statistical Analysis

Baseline characteristics were compared between two groups (complex and noncomplex WOPFCs) by using *χ*^2^ test for categorical variations, Student's *t*-test for continuous variations, and Mann–Whitney *U*-test for nonparametric variation. MANOVA was used for multivariate analysis. SPSS software version 22.0 for Windows (IBM Corp., Chicago, IL, USA) was used for performing statistical analyses.

## 3. Results

A total of 31 patients with WOPFCs which underwent EUS-PFC with LAMS were recruited. Infected WOPFC was the indication for EUS-PFC in all patients. The mean age of these patients was 48 ± 19 years. Nineteen patients (61.3%) were male. Underlying chronic pancreatitis was found in seven patients (22.6%). For etiologies of pancreatitis, gallstone pancreatitis is the most common one (*n* = 13, 41.9%), followed by alcoholic pancreatitis and other etiologies (drug-induced, idiopathic pancreatitis) at 32.3% and 25.8%, respectively. Twenty-one of 31 (67.7%) had walled-off necrosis (WON). All WON patients required DEN.

**Figure 1:**
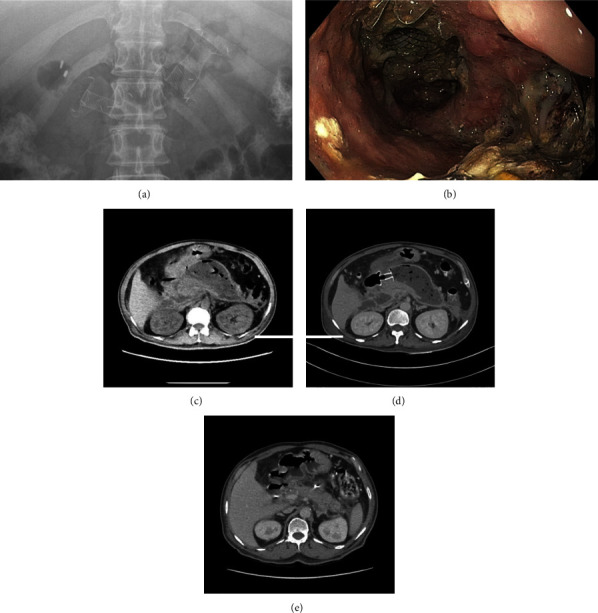
Patients in group A. (a) Two LAMSs were inserted into WON. (b) The first LAMS was seen inside WON during DEN via the second LAMS. (c) CT scan showed an air-bubble containing WON which indicated infection. (d) CT scan after first LAMS insertion and DEN, WON size was not decreased. (e) CT scan after both LAMSs were removed, the previous WON was resolved. (Abbreviation, LAMS; lumen-apposing metal stent, WON; walled-off necrosis, DEN; direct endoscopic necrosectomy).

Six of 31 (19.4%) patients required multiple drainage procedures than a single LAMS. They were classified as group A. All six patients in group A had WON and required DEN. Five of six patients in group A were indispensable to subsequent percutaneous drainage. The other patient required a second LAMS to complete drainage because of highly viscous collecting fluid; two LAMSs were placed on by trans-duodenal and trans-gastric approach (Figure 1). Patients in group A tended to require more DEN sessions than group B (Median 3.5 (range 1–13) versus 0 (range 0–8) times, *p* = 0.06). The maximum DEN session was 13 times in a patient with large multiloculated WON with candida infection. Out of six patients in group A, pancreatic duct (PD) leakage was found in four patients (66.7%), which were treated by plastic stent placement.

**Figure 2:**
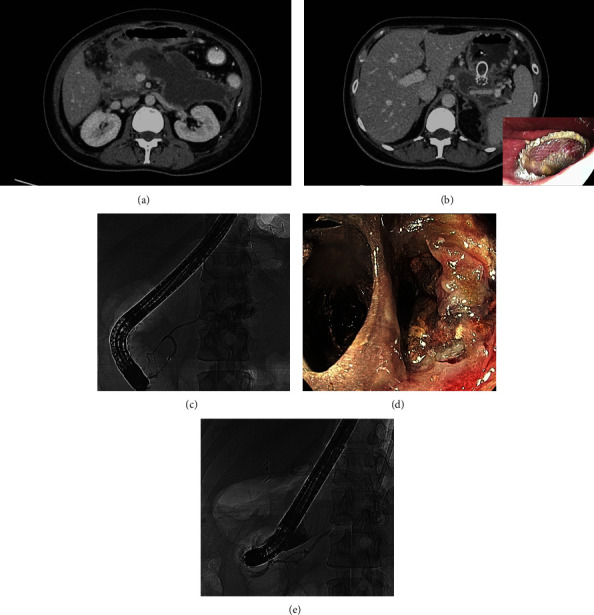
Patient in group B. (a) CT scan showed a 14-centimeter WON. (b) A LAMS was inserted into WON (c) Pancreatogram showed pancreatic duct leakage at the body of the pancreas, which were treated with plastic stent. (d) Necrotic debris and fibrous septum were seen during DEN, which were removed. (e) Follow-up pancreatogram showed resolution of pancreatic duct leakage. (Abbreviation, LAMS; lumen-apposing metal stent, WON; walled-off necrosis, DEN; direct endoscopic necrosectomy).

In group B (*n* = 25), 15 patients (60.0%) had WON which required DEN (Figure 2) in 11 patients (73.3%). In the other 10 patients with pseudocyst, DEN was required in only one patient because it contained a solid component. The maximum DEN session in group B was eight times in a WON patient with a large number of necrotic debris. PD leakage was found in five patients (20.0%).

From the univariate analysis of patients' characteristics, the maximal diameter of WOPFCs in group A was significantly larger than group B (18 ± 6 versus 13 ± 3 cm in diameter, respectively, *p* = 0.021). Solid component proportion was higher in group A (35.8% versus 17.8%, respectively, *p* = 0.025). the presence of PD leakage was higher in group A versus B (67% versus 20%, respectively, *p* = 0.025). No statistical difference between both groups regarding age, sex, percentage of WON, etiology of pancreatitis, baseline chronic pancreatitis, and pelvic and paracolic gutter extension was seen as shown in Table 1.

A univariate analysis of procedural information (Table 2) showed that the need of DEN was significantly higher in group A versus B (100% versus 48%, respectively, *p* = 0.020). The median number of DEN sessions was also higher in group A versus B (3.5 versus 0 time (s), respectively, *p* = 0.041). No difference was seen between using small versus large-diameter LAMS.

Sequential multivariate analysis showed a similar result to the univariate analysis: the maximal diameter of WOPFCs in group A was significantly larger than group B (*p* = 0.021). Solid component proportion was higher in group A (*p* = 0.025). The incidence of PD leakage was higher in group A (*p* = 0.021). The need of DEN and the number of DEN sessions was higher in group A (*p* = 0.020 and 0.031 respectively). No statistical difference between both groups in age, sex, percentage of WON, etiology of pancreatitis, baseline chronic pancreatitis, and resolution time of WOPFCs was observed.

After receiving adequate debridement and LAMS removal, all patients had complete resolution of WOPFCs without any recurrence during the 6-month follow-up period. Minor adverse events occurred in two patients in group A, that did not affect treatment outcomes. In the first patient, stent migration occurred during the first DEN session. However, new LAMS was successfully inserted, and DEN was continued. In the second patient, LAMS was deployed into the WON cavity. Fortunately, new LAMS was used and properly deployed. The misdeployed LAMS was safely retrieved during the DEN procedure through the cystogastrostomy opening. There was no bleeding associated with LAMS. During the 6-month follow-up, PFC recurrence and death did not occur.

## 4. Discussion

The current study is a multicenter retrospective study from three large referral hospitals aiming to compare different clinical characteristics of WOPFCs treated with LAMS (s) plus additional drainage methods versus only single LAMS. The current study showed significant differences in several parameters between both groups including more complexity of lesions plus higher number of DEN procedures to accomplish the drainage procedure. This kind of information could be used to predict the prognosis of WOPFCs and categorize WOPFCs into a group requiring LAMS (s) plus other drainage procedures or single LAMS for EUS-PFC.

The paradigms of drainage of WOPFCs have been changed from surgery/percutaneous drainage to endoscopic drainage, particularly EUS-PFC in the last decade [2]. Stents used for drainage varied from plastic stents, tubular self-expandable metal stents, or LAMS. A large-diameter metal stent is practically preferred over the plastic stent because of its higher treatment success rate and lower number of procedures [10–12]. However, a recent randomized controlled trial and meta-analysis showed a conflicting result that LAMS was not superior than the plastic stent in terms of treatment outcome [13, 14]. Nevertheless, the American Gastroenterological Association still recommends EUS-guided LAMS drainage for centrally located WON. In this present study, we focus on WOPFCs that were treated with LAMS, and results showed that WOPFCs with more solid component, bigger size of lesions, and pancreatic duct leakage required additional drainage method, higher number of DEN procedures to accomplish EUS-PFC.

Currently, LAMSs provide effective drainage for WOPFCs. With LAMS, early DEN could be performed to promote the resolution rate of WOPFCs up to 91.3% [7]. However, the present study showed that only 81% of WOPFCs patients achieved adequate drainage with single LAMS. This can be explained by several reasons including different criteria to start the DEN procedure for WOPFCs in different centers, or different local resources in each institution, or different complementary techniques such as the insertion of naso-biliary tubes with or without irrigation [15]. In condition that WOPFCs could not be resolved by EUS-PFC alone (e.g., large WOPFC with multiple recess and ongoing PD leakage) for additional drainage, the percutaneous route might be the drainage of choice [16]. This finding is similar to results from the present study that half of the patients with paracolic gutter extension required percutaneous drainage.

In addition to percutaneous drainage, transcutaneous endoscopic necrosectomy (TEN) could be added on in needed cases [17]. Saumoy et al. [18] performed percutaneous drainage followed by TEN by using the esophageal stent as the portal and reported 89% clinical success rate of WOPFCs resolution. The closure of the fistula tract subsequently succeeded in all patients with clinical success. Anyway, patients with WOPFCs that could be accessed by second EUS-PFC and second LAMS should be firstly considered as EUS-PFC in an internal drainage procedure. The second LAMS placement might also be helpful in the condition that fluid in WOPFCs had high viscosity with or without necrotic debris. Two LAMSs allowed air to go inside the WOPFCs to maintain positive pressure and pushed necrotic debris through the other. This is the concept named as the multiple gateway technique [19]. In this current study, out of six patients requiring additional drainage, five had additional percutaneous drainage, and one had another LAMS to accomplish EUS-PFC.

It is still not sure what size of LAMS is appropriate for the EUS-PFC procedure or WOPFCs. Parsa et al. [20] showed a comparable clinical success rate between 15-mm LAMS and 20-mm LAMS. These assumed that 10-mm LAMS or lager are sufficient for WOPFC drainage. However, results from this present study showed that a 10-mm LAMS was not inferior to 15 or 16-mm LAMS for drainage adequacy. Nevertheless, the number of patients in this present study is very small for such a conclusion. In our experience, we would recommend LAMS with diameter larger than 10 mm as LAMS of choices for EUS-PFC particularly in WOPFCs with features similar to group A patients as it may be convenient to insert a gastroscope for subsequent DEN.

DEN plays an important role for management of WOPFCS [21]. Gardner et al. [6] demonstrated that DEN dramatically improved the resolution rate of WON (88% compared to 45% in endoscopic transmural drainage alone). Mechanical debridement is performed by different devices such as biopsy forceps, rat-tooth forceps, and snare. Recently developed endoscopic morcellator devices can rapidly liquefy the necrotic debris [22]. Hydrogen peroxide is also the option for chemical debridement. It liquefied the necrotic debris and facilitated mechanical debridement [23]. Results from this current study showed a higher number of DEN sessions in group A which had more complex features of lesion. This supported use of DEN to resolve complex WOPFC and might indirectly shorten time to resolution of WOPFCS; however, the later assumption could not be analyzed from the result of this current study.

This present study, on the other hand, illustrated good outcomes of the WOPFCs with step-up endoscopic management. Neither mortality nor serious adverse events were found during study time. WOPFCs resolution time was not different among both groups. This can be implied that the early step-up approach with early DEN and additional drainages improve these outcomes. A recent large study showed good results of EUS-PFC with step-up policy [24].

Postpancreaticobiliary procedural complications such as post-ERCP pancreatitis could be considered as preventable causes of pancreatitis. Not only rectal indomethacin should be used [25] but also the advanced bile duct cannulation technique including the double-guidewire technique, early precut sphincterotomy, and endoscopic ultrasound-guided rendezvous could be helpful in case of difficult bile duct access [26]. The postoperative fluid collections (POFC) following pancreaticobiliary surgery are similar to WOPFC. The endoscopic ultrasound-guided drainage showed a higher clinical success rate compared to percutaneous drainage. However, data on endoscopic ultrasound-guided drainage with LAMS were limited [27].

A practical benefit from this study is that the knowledge can be used to classify the type of WOPFCs and how it should be treated for the most benefit of the patient. The strength of this study was that there was little information on the subject of classification of EUS-PFCs for the purpose of prognosis and preparation for treatment methods. The weakness of this study was the small number of patients. However, the total number of patients was patients with significant clinical symptoms until the patient had to undergo EUS-PFCs with LAMS.

In summary, this is an interesting study, although the number of cases is small, a part of the reason for the low number of cases was because the investigators intended to select only patients with severe symptoms requiring LAMS for treatment. The researchers were able to show that patients requiring LAMS in combination with other methods had a different radiographic profile than those who underwent LAMS alone. The results of this study might be applied in clinical practice by categorizing patients into groups and allowing the endoscopist to predict a more accurate prognosis which would lead to the determination of appropriate treatment approach.

## Figures and Tables

**Table 1:** Baseline characteristics of the patients. Patients with WOPFCs requiring LAMS plus additional drainage methods including additional LAMS, percutaneous drainage, or surgical debridement were categorized as “complex WOPFCS” or classified as group A whereas ones requiring only single were grouped as “noncomplex WOPFCS” or group B.

Baseline characteristics (*n* = 31)	Group A (*n* = 6)	Group B (*n* = 25)	Univariate *p* value	Multivariate *p* value
Age, years (SD)	42 (19)	49 (19)	0.548	0.388
Male sex, *n* (%)	3 (50)	16 (64)	0.527	0.543
Walled-off necrosis, *n* (%)	6 (100)	15 (60)	0.060	0.063
Mean estimated solid component proportion (%)	17.8	35.8	0.025 ^*∗∗*^	0.025 ^*∗∗*^

Etiology of pancreatitis *n* (%)			0.364	0.725
Alcoholic pancreatitis	3 (50)	7 (28)		
Gallstone pancreatitis	1 (17)	12 (48)		
Others	2 (33)	6 (24)		

Chronic pancreatitis, *n* (%)	0 (0)	7 (28)	0.141	0.150
Pancreatic duct leakage, *n* (%)	4 (67)	5 (20)	0.024 ^*∗∗*^	0.023 ^*∗∗*^
Size of collection, cm (SD)	18 (6)	13 (3)	0.021 ^*∗∗*^	0.021 ^*∗∗*^
Presence of paracolic gutter extension, *n* (%)	3 (50)	7 (28)	0.301	0.317
Presence of pelvic extension, *n* (%)	2 (33)	2 (8)	0.096	0.103

**Table 2:** Procedural data and outcomes. Patients with WOPFCs requiring LAMS plus additional drainage methods including additional LAMS, percutaneous drainage, or surgical debridement were categorized as “complex WOPFCS” or classified as group A whereas ones requiring only single were grouped as “noncomplex WOPFCS” or group B.

Procedural data and outcomes (*n* = 31)	Group A (*n* = 6)	Group B (*n* = 25)	Univariate *p* value	Multivariate *p* value
Small-diameter LAMS, *n* (%)	4 (67)	8 (32)	0.117	0.125
DEN, *n* (%)	6 (100)	12 (48)	0.020 ^*∗∗*^	0.020 ^*∗∗*^
Median DEN sessions (range)	3.5 (1–13)	0 (0–8)	0.041 ^*∗∗*^	0.031 ^*∗∗*^
Resolution time, days (SD)	27 (23)	25 (16)	0.898	0.561

Adverse event, *n* (%)	2 (50)	0 (0)	N/A	N/A
Stent migration, *n* (%)	1 (25)	0 (0)		
Stent mis-deployment, *n* (%)	1 (25)	0 (0)		

## Data Availability

Dataset used and analyzed to support this study are available from the corresponding author upon request.

## Abbreviation

DEN: Direct endoscopic necrosectomy
EUS: Endoscopic ultrasound
EUS-PFC: EUS-guided drainage of symptomatic walled-off pancreatic fluid collection
LAMS: Lumen-apposing metal stent
PD: Pancreatic duct
TEN: Transcutaneous endoscopic necrosectomy
WON: Wall-off necrosis
WOPFC: Walled-off pancreatic fluid collection.
